# The Efficiency, Predictability, and Safety of First-Generation (Visumax 500) and Second-Generation (Visumax 800) Keratorefractive Lenticule Extraction Surgeries: Real-World Experiences

**DOI:** 10.3390/life14070804

**Published:** 2024-06-26

**Authors:** Chia-Yi Lee, Ie-Bin Lian, Hung-Chi Chen, Chin-Te Huang, Jing-Yang Huang, Shun-Fa Yang, Chao-Kai Chang

**Affiliations:** 1 Institute of Medicine, Chung Shan Medical University, Taichung 402, Taiwan; 2 Nobel Eye Institute, Taipei 115, Taiwan; 3 Department of Ophthalmology, Jen-Ai Hospital Dali Branch, Taichung 402, Taiwan; 4 Institute of Statistical and Information Science, National Changhua University of Education, Changhua 500, Taiwan; 5 Department of Ophthalmology, Chang Gung Memorial Hospital, Linkou 333, Taiwan; 6 Center for Tissue Engineering, Chang Gung Memorial Hospital, Linkou 333, Taiwan; 7 Department of Medicine, Chang Gung University College of Medicine, Taoyuan 333, Taiwan; 8 Department of Ophthalmology, Chung Shan Medical University Hospital, Taichung 402, Taiwan; 9 Department of Ophthalmology, School of Medicine, Chung Shan Medical University, Taichung 402, Taiwan; 10 Department of Medical Research, Chung Shan Medical University Hospital, Taichung 402, Taiwan; 11 Department of Optometry, Da-Yeh University, Chunghua 515, Taiwan

**Keywords:** keratorefractive lenticule extraction, visumax 800, smile pro, uncorrected distance visual acuity, spherical equivalent

## Abstract

We aimed to evaluate the postoperative visual and refractive outcomes between the first- and second-generation keratorefractive lenticule extraction (KLEx) surgeries. A retrospective cohort study was conducted and patients who had received first- and second-generation KLEx surgeries were enrolled. A total of 80 and 80 eyes were categorized into the first and second KLEx groups after exclusion, respectively. The primary outcomes were the postoperative uncorrected distance visual acuity (UDVA), spherical equivalent (SE), and safety indexes. An independent *t*-test and generalized estimate equation were implemented to compare the primary outcomes between the two groups. After the KLEx surgery, the UDVA showed no significant difference between the two groups throughout the study period (all *p* > 0.05), and the postoperative SE and safety index were also statistically identical between the two groups during the follow-up interval (all *p* > 0.05). There was a similar trend of visual recovery between the two groups (aOR: 0.967; 95% CI: 0.892–1.143; *p* = 0.844), while the amplitude of the SE change was significantly lower in the second KLEx group (aOR: 0.760; 95% CI: 0.615–0.837; *p* = 0.005). Nine and two unintended initial dissection of the posterior plane (UIDPP) occurred intraoperatively in the first and second KLEx groups, respectively, and the second group showed a lower risk of UIDPP (*p* = 0.032). In conclusion, the efficiency, predictability, and safety are similar between first- and second-generation KLEx surgeries.

## 1. Introduction

Corneal refractive surgeries have been used to correct refractive errors, including myopia, astigmatism, and hyperopia, for decades [1,2]. Laser in situ keratomileusis and photorefractive keratectomy have been applied for more than 20 years, and their visual outcomes are acceptable [2]. A postoperative uncorrected distance visual acuity (UDVA) of 20/20 has been reported in 92% of patients who received laser in situ keratomileusis and in 72% of patients who received photorefractive keratectomy [3,4]. Still, several postoperative complications, including persistent ocular irritation, prominent corneal nerve damage, and dry eye disease, have been demonstrated in the two above-mentioned corneal refractive surgeries, which may reduce patient satisfaction [5].

Keratorefractive lenticule extraction (KLEx), previously known as small incision lenticule extraction [6], is a corneal refractive surgery first introduced around 2010, in which a corneal lenticule made of a femtosecond laser is extracted to reduce myopia and astigmatism [7,8,9]. Compared with earlier corneal refractive surgeries, KLEx has the advantage of a small incision, which contributes to lesser postoperative dry eye disease [10,11]. Regarding the visual and refractive outcomes, first-generation KLEx has been shown to be comparable to both laser in situ keratomileusis and photorefractive keratectomy in previous articles [12,13,14,15]. In addition, postoperative astigmatism and higher-order aberrations of first-generation KLEx surgery showed not-inferior values compared with laser in situ keratomileusis, although no wavefront-guided function is available in KLEx surgery [16,17].

Last year, the second generation of KLEx was announced in the field of corneal refractive surgeries [18]. Compared with first-generation KLEx, second-generation KLEx has a faster laser emission speed and the presence of an eye-tracking system [19,20]. However, there is scant research comparing the first- and second-generation KLEx surgeries. Because of the modifications of the laser device and software in the second-generation KLEx, the surgical outcomes between them may be different, which needs further evaluation. This issue should be investigated because second-generation KLEx surgery is a refractive surgery that has been available to the public for just two years [18], and there is little research discussing the outcomes of this surgery and the comparison between it and other refractive surgeries. Since second-generation KLEx surgery has recently gained popularity (at least in Taiwan and China), the evaluation of this surgery cannot be overemphasized.

Consequently, the objective of the current study was to investigate the visual (the efficiency) and refractive (the predictability) outcomes of first- and second-generation KLEx surgeries within a three-month interval. The safety index and any intraoperative/postoperative complications were also recorded and analyzed. The primary outcomes of the current study were the postoperative UDVA, spherical equivalent (SE), and safety indexes.

## 2. Materials and Methods

### 2.1. Participant Selection

This retrospective cohort study was executed at the Nobel Eye Institute, which has several branches in Northern, Central, and Southern Taiwan. Patients were included in the current study if they aligned with the following factors: (1) aged from 20 to 55 years, (2) presented with a cycloplegia sphere power of more than −1.00 diopter (D) but lower than −10.00 D for first-generation KLEx surgery and −9.00 D for second-generation KLEx surgery, (3) received first-generation or second-generation KLEx surgery at the Nobel Eye Institute, and (4) continuously visited any branch of the Nobel Eye Institute after the surgery for at least three months. The patients were scheduled for first-generation or second-generation KLEx surgery according to their choice after a thorough discussion with the ophthalmologists. If a patient was ordered to undergo first- or second-generation KLEx surgery with monovision (planning residual myopia) but their actual sphere power was higher than the inclusion criteria, the patient was not to be included in the current study. The following exclusion criteria was used to erase the patients with an extremely poor preoperative condition: (1) a best corrected visual acuity (BCVA) lower than 20/40; (2) the presence of severe corneal or retinal diseases, including, but not limited to, central corneal opacity, keratoconus, proliferative diabetic retinopathy, macula-off retinal detachment and central retinal vein occlusion; (3) the presence of uncontrolled glaucoma or uveitis; (4) a change in refraction for more than 0.50 D in the previous year; and (5) pregnancy status in the last three months. Moreover, we chose the eye to be included and analyzed by drawing lots. After the whole process, a total of 80 and 80 eyes were included into the first and second KLEx groups, respectively.

### 2.2. Surgery Technique

All the KLEx procedures in the current study were performed by two experienced refractive specialists (C.-Y.L. and C.-K.C.). The procedure was completed using a first-generation femtosecond laser device (Visuamax 500, Carl Zeiss, Göschwitzer Str., Jena, Germany) and a second-generation femtosecond laser device (Visuamax 800, Carl Zeiss, Göschwitzer Str., Jena, Germany). For the first-generation KLEx surgery, the optic zone ranged from 5.5 to 6.9 mm according to the ablation depth and pupil size, and the corneal incision was created as 3.0 mm at 105 degrees. After the angle kappa was confirmed with a microscope, with the aid of corneal topography and the coaxial sighted corneal light reflex, the whole cornea was fixated using a suction ring. After the femtosecond laser emission, a specialized spatula was employed to separate the upper and lower interface of the lenticule, and then the lenticule was removed with forceps. For the second-generation KLEx surgery, the surgical steps were largely identical to the first-generation KLEx surgery except that the angle kappa was illustrated by the software in accordance with data obtained from optical biometry (IOL Master 700, Carl Zeiss, Göschwitzer Str., Jena, Germany). After the surgery, levofloxacin eye drops and prednisolone eye drops were instilled for about one week, then switched to sulfamethoxazole and fluorometholone eye drops for another three weeks. Artificial tears were utilized for two months after the surgery.

### 2.3. Ophthalmic Exam

All the patients receiving the KLEx surgery accepted identical ophthalmic exams at any branch of the Nobel Eye Institute. The preoperative exams included BCVA by manifest refraction, cycloplegia refraction of the sphere power and cylinder power using an autorefractor (KR-8900, Topcon, Itabashi-ku, Tokyo, Japan), central corneal thickness (CCT), corneal astigmatism, pupil diameter using a topographic machine (TMS-5, Tomey Coporation, Nagoya, Aichi, Japan), and axial length (AXL) using a biometry machine (IOL Master 700, Carl Zeiss, Göschwitzer Str., Jena, Germany). The postoperative examinations involved UDVA, BCVA, and sphere and cylinder powers by manifest refraction. The postoperative exams were conducted via devices identical to those used for preoperative exams. Additionally, the side-cut depth, optic zone, cap thickness, residual stromal thickness (RST) and lenticule thickness of KLEx surgery were collected. The data before, one day after, one week after, one month after, and three months after the KLEx surgery were recorded. Importantly, the SE was determined as the sphere power plus half of the cylinder power in the current study, and postoperative dry eye disease was defined as preoperative tear secretion of more than 10 mm and the presentation of prominent dryness after the KLEx surgery.

### 2.4. Statistical Analysis

SPSS version 20.0 (SPSS Inc., Chicago, IL, USA) was implemented for the statistical analysis. The statistical power of the current study was 0.91 with a 0.05 alpha value and a medium effect size which was generated using G∗power version 3.1.9.2 (Heinrich Heine Universität Düsseldorf, Düsseldorf, Germany). The Shapiro–Wilk test was implemented to inspect the normality of the study population and a normal distribution was found (*p* > 0.05). A descriptive analysis was implemented to demonstrate the age, sex, refraction status, topographic parameters, and surgical parameters between the two groups, and an independent *t*-test was implemented to examine these factors between the two groups. The independent *t*-test was also implemented to assess the efficiency (UDVA), predictability (SE), and safety (postoperative BCVA divided by preoperative BCVA) between the first KLEx and second KLEx groups at different time points. For the UDVA and SE change trends in the follow-up period, the generalized estimate equation was implemented to examine the difference between the first KLEx and second KLEx groups with adjustments for age, sex, and preoperative refractive status; then, the adjusted odds ratio (aOR) with a 95% confidence interval (CI) for UDVA and SE between the two groups was presented. The sex was regarded as a confounding factor in the multivariable analysis for the UDVA and SE trends between the two groups. A line chart was used to display the change in UDVA and SE after the KLEx surgery. The prominent intraoperative and postoperative complications were also collected, and Fisher’s exact test was implemented to examine the ratio of complications between the two groups. Finally, the medical charts of each patient were reviewed three months postoperatively, and negative words such as “blurry vision”, “poor visual quality”, “severe halo”, or “severe visual disturbance” in the subjective column were recorded as dissatisfaction. Then, Fisher’s exact test was implemented again to compare the rate of dissatisfaction between the two groups. A *p* value < 0.05 was determined as statistical significance. A *p* value over 0.999 was displayed as *p* > 0.999, while a *p* value under 0.001 was displayed as *p* < 0.001 in the current study.

## 3. Results

The baseline parameters of the study population are exhibited in Table 1. The mean age was 31.52 ± 7.42 and 33.38 ± 8.87 years old in the first KLEx group and second KLEx group, respectively. The difference in age was not significant between the two groups (*p* = 0.152). In addition, the distribution of sex was also similar between the first and second KLEx groups (*p* = 0.426). The second KLEx group showed a higher ratio of systemic disease (*p* = 0.017), while other preoperative parameters were statistically identical between the first and second KLEx groups (all *p* > 0.05) (Table 1).

**Figure 1:**
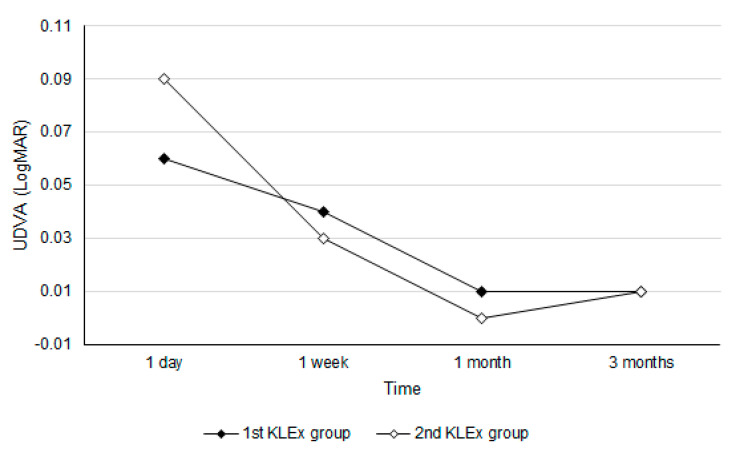
The trends of the uncorrected distance visual acuity between the two groups. UDVA: uncorrected distance visual acuity.

**Figure 2:**
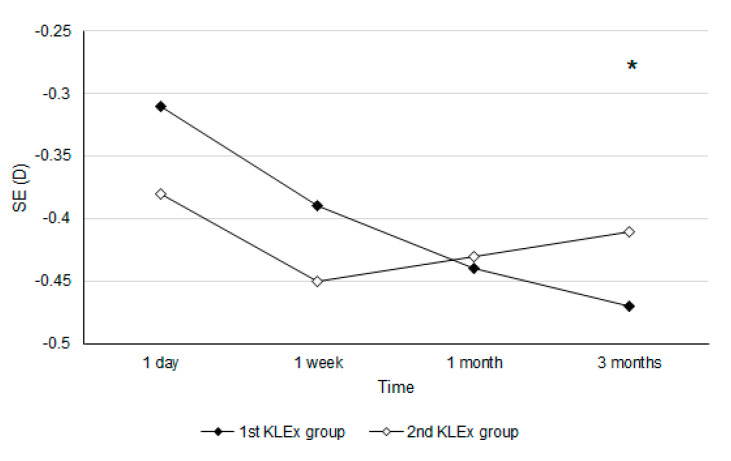
The trends of the spherical equivalent between the two groups. D: diopter, SE: spherical equivalent. * denotes significant difference between the two groups.

After the KLEx surgery, the UDVA one day postoperatively showed no significant difference between the two groups (0.06 ± 0.08 vs. 0.09 ± 0.19, *p* = 0.196), and the difference in the UDVA between the two groups remained statistically insignificant throughout the study period (all *p* > 0.05) (Table 2). For the refraction aspect, the SE one day postoperatively was also statistically identical between the two groups (−0.31 ± 0.51 vs. −0.38 ± 0.52, *p* = 0.391) and persisted throughout the whole study period (all *p* > 0.05) (Table 2). Concerning the safety of KLEx, the safety index three months postoperatively was 1.02 and 1.00 in the first and second KLEx groups, which showed no significant difference (*p* = 0.826). The trend analysis showed a similar trend of visual recovery between the two groups (aOR: 0.967; 95% CI: 0.892–1.143; *p* = 0.844) (Figure 1), while the amplitude of SE change was significantly lower in the second KLEx group (aOR: 0.760; 95% CI: 0.615–0.837; *p* = 0.005) (Figure 2). The male sex showed a better UDVA improvement compared to the females (aOR: 1.252, 95% CI: 1.017–1.533, *p* = 0.026), but the change in SE in the males did not show significant difference compare to the females (aOR: 1.045, 95% CI: 0.865–1.340, *p* = 0.529).

Nine and two unintended initial dissection of the posterior plane (UIDPP) occurred intraoperatively in the first KLEx group and the second KLEx group, respectively, and the second group showed a lower risk of UIDPP than the first group (*p* = 0.032). The incidence of other intraoperative complications, including incision tears, cap perforation, residual lenticule and suction loss, did not demonstrate significant differences between the two groups (all *p* > 0.05) (Table 3). On the other hand, the risks of all the postoperative complications, including superficial punctate keratitis, dry eye disease, corneal edema, interface foreign body, epithelial ingrowth, diffuse lamellar keratitis, and microbial keratitis, did not show significant differences between the two groups (all *p* > 0.05) (Table 3). There were three (3.75%) and two (2.50%) dissatisfaction events in the first KLEx group and second KLEx group, respectively, and the difference was statistically insignificant (*p* = 0.689).

## 4. Discussion

The postoperative UDVA between the first- and second-generation KLEx surgeries did not reveal significant differences. Moreover, the refraction status was similar between the first- and second-generation KLEx surgeries, while patients who received second-generation KLEx surgery underwent a lower amplitude of refraction change. On the other hand, the safety index and complications were statistically identical between the two surgeries except that the second-generation KLEx surgery demonstrated a lower risk of UIDPP episodes. Because the postoperative UDVA and refractive status indicate the efficiency and predictability of a refractive surgery, the efficiency and predictability of the first and second KLEx surgeries are comparable. Additionally, the safety index is acceptable in both groups, and the UIDPP events, which are a complication that does not influence the long-term surgical outcome, showed a higher rate in the first KLEx group. Thus, the overall safety of the two surgeries may be similar.

The postoperative UDVA was similar between the first-generation and second-generation KLEx surgery in the current study. In a previous study, 95.4% of patients achieved a UDVA of 20/20 twelve months after the first-generation KLEx surgery [7]. For the second-generation KLEx surgery, one study revealed that a postoperative UDVA of 20/20 was reached in 91% of eyes three months after the surgery [19]. However, there is scant research that surveys the postoperative UDVA between the first- and second-generation KLEx surgeries in the same population that live in nearby regions. To our knowledge, the results in the current study may be a preliminary experience to demonstrate the similar efficiency of first-generation and second-generation KLEx surgery. Furthermore, the baseline demographic data and refraction status were similar between the first KLEx group and the second KLEx group; thus, the homogeneity of the study group might be adequate. The difference in the three-month-postoperative UDVA between the first KLEx group and the second KLEx group was minimal, which is an insignificant value in both the statistical and clinical aspects and demonstrates an identical efficiency between the first- and second-generation KLEx surgeries after the whole recovery period. Concerning the trend of visual recovery between the first- and second-generation KLEx surgeries, the curve in the UDVA value did not illustrate a significant difference between the two groups. Although the second-generation KLEx surgery applied more laser frequency and spots compared with the first-generation KLEx surgery [19,20], the similar postoperative UDVA recovery may indicate that the laser energy is within the tolerable range for the human cornea. Moreover, the shorter suction time in second-generation KLEx surgery may cause lesser corneal epithelial injury and benefit visual recovery [20]. The male sex showed a better visual recovery than the female population, which is similar to the previous study where the males correlated to a higher efficiency of the refractive surgery [21].

Regarding the predictability of postoperative refraction, the postoperative SEs at different time points were statistically identical between the first KLEx group and the second KLEx group. The postoperative mean SE of the first-generation KLEx surgery ranged from −0.13 to −0.22 D [7,22], which is a fair value compared with other refractive surgeries including laser in situ keratomileusis and photorefractive keratectomy [3,4]. In addition, previous research reported that 86% of patients who received second-generation KLEx surgery had an SE within ±0.50 D after a three-month period [19]. In the current study, the similar postoperative SE between the first KLEx group and the second KLEx group further illustrated the similar predictability between the first- and second-generation KLEx surgeries in populations with similar preoperative refractive status. In terms of astigmatism, a numerically smaller cylinder power was observed in the second KLEx group compared with the first KLEx group, and both groups showed similar residual astigmatism values (about −0.30 D) compared to previous studies [23,24,25]. This may imply that postoperative astigmatism was insignificantly lower in those who received the second-generation KLEx surgery than in those who received the first-generation KLEx surgery. For the trend in the SE change between the two groups, the second KLEx group presented a significantly lower change in SE than the first KLEx group, in which a silent increment in the SE was observed in the first KLEx group. There are few studies that present this phenomenon. A possible explanation is that the smooth interface of second-generation KLEx surgery resulted from the high-density laser emission contributing to a higher regularity of the whole cornea thus increasing the refractive stability in the second-generation KLEx surgery. Still, the change in the SE throughout the study period was only −0.16 and −0.03 in the first and second KLEx groups, which is clinically insignificant; thus, the predictability of both KLEx surgeries is, in fact, close. The change in SE did not show significant difference between the two groups. In a previous study discussing photokeratorefractive surgery, the males and females presented with a similar trend of refractive error regression [26]. As a consequence, the results of our study could correspond to previous experience.

The safety index can be applied to judge the possibility of visual loss in corneal refractive surgery [21], and the safety index did not show a significant difference between the two KLEx groups in the current study. There were two and two eyes with decreased BCVA values in the first and second KLEx groups, respectively. When compared with the safety indexes of KLEx surgery in previous studies, the safety indexes of the first and second KLEx groups in the current study are acceptable [7,19,22]. Moreover, the four eyes with reduced BCVA only lost one line of BCVA on the Snellen chart, and no eye lost more than two lines of BCVA in the current study. Consequently, the extent of BCVA loss in both the first KLEx and second KLEx groups might not be dominant. On the other hand, the intraoperative as well as postoperative complications after corneal refractive surgery can influence both visual acuity and corneal health [27,28]. UIDPP during KLEx surgery causes a longer operation time and worse initial UDVA, while the final UDVA and SE are similar to those with smooth KLEx surgery [29]. The lower rate of UIDPP in the second KLEx group may not indicate a better postoperative outcome, but the faster recovery and shorter operation time due to fewer UIDPP events may benefit patients’ satisfaction with the second-generation KLEx surgery. The other intraoperative and postoperative complications did not demonstrate significant differences between the first KLEx and second KLEx groups, which indicates that the overall safety between the two surgeries is compatible.

Concerning the postoperative outcomes of the second-generation KLEx surgery in the current study compared with other refractive surgeries in previous studies, the UDVA of the second-generation KLEx surgery was 0.01, which is similar to the UDVA of laser in situ keratomileusis in an earlier publication [30]. Additionally, the three-month-postoperative UDVA of the second-generation KLEx surgery was numerically better than that of photorefractive keratectomy [4]. Regarding the postoperative refraction, 99% of patients who received the second-generation KLEx surgery had a postoperative SE within ±1.00 D, which is similar to the refractive outcomes of patients who received laser in situ keratomileusis and the first-generation KLEx surgery [22,30]. In addition, the safety index of the second-generation KLEx surgery was 1.00, while the safety index of laser in situ keratomileusis was 1.06 in the previous literature [31]. If we compare the efficiency, predictability, and safety of the second-generation KLEx surgery in the current study and in the previous studies, the results of the current study are still comparable to previous experiences [18,19]. On the other hand, the postoperative outcomes of the first-generation KLEx surgery in the current study were also compatible with those of the first-generation KLEx surgery and other corneal refractive surgeries in preceding studies [7,12]. These results indicate that the quality of both the first- and second-generation KLEx surgeries may be acceptable in our institution.

There are some limitations of the current study. Firstly, the retrospective design of the current study reduced the homogeneity of the study population, although no significant difference was found regarding the preoperative parameters between the first KLEx and second KLEx groups. In addition, the total number of eyes was relatively insufficient; only 150 eyes were enrolled in the current study, which may cause statistical bias. In addition, not all the operations were performed by a single surgeon. Although the same protocol was applied, the surgical technique may still be slightly different between different surgeons. Finally, only the preoperative topographic and biometry parameters were collected due to the retrospective design of the current study; thus, some crucial comparisons, such as the topographic changes after KLEx surgery, cannot be assessed.

## 5. Conclusions

In conclusion, the efficiency and predictability between the first- and second-generation KLEx surgeries were statistically identical in patients with similar preoperative conditions. Furthermore, the safety index and surgical-related complications between the two surgery types were also similar. Consequently, the second-generation KLEx surgery has the advantages of a faster surgical time and an eye-tracking function compared with the first-generation KLEx surgery and can be recommended to patients with a higher degree of anxiety or a large angle kappa, which makes it harder to guide the eyeball. On the other hand, if the patients did not present with an anxiety status or a large angle kappa, both KLEx surgeries could be chosen due to the similar postoperative outcomes. For naïve refractive surgeons, the lower risk of UIDPP in second-generation KLEx surgery could shorten the learning curve. Further large-scale prospective studies to evaluate the efficiency and predictability between first- and second-generation KLEx surgeries in specific populations are required.

## Figures and Tables

**Table 1:** Basic characteristics of the two groups.

Characteristics	First KLEx Group (N = 80)	Second KLEx Group (N = 80)	*p*
Age	31.52 ± 7.42	33.38 ± 8.87	0.152
Sex (male/female)	38:42	32:48	0.426
Laterality (right/left)	36:44	39:41	0.535
Systemic disease			0.017 *
Hypertension	0	6	
Diabetes mellitus	0	2	
Heart disease	2	4	
Others	3	6	
BCVA (LogMAR)	0.00 ± 0.02	0.01 ± 0.04	0.154
Manifest refraction (D)			
Sphere	−4.75 ± 2.11	−4.93 ± 2.06	0.597
Cylinder	−0.98 ± 0.93	−1.12 ± 1.02	0.385
SE	−5.24 ± 2.26	−5.49 ± 2.31	0.503
Cycloplegic refraction (D)			
Sphere	−4.78 ± 1.93	−4.83 ± 2.01	0.873
Cylinder	−1.01 ± 1.00	−1.16 ± 1.05	0.356
SE	−5.28 ± 2.04	−5.40 ± 2.28	0.715
Topographic cylinder (D)	1.44 ± 0.83	1.49 ± 0.67	0.668
CCT (μm)	553.50 ± 27.74	554.98 ± 38.93	0.783
Pupil diameter (mm)	3.91 ± 0.70	3.78 ± 0.59	0.198
Schirmer test (mm)	13.74 ± 8.19	15.24 ± 7.22	0.219
Optic zone (mm)	7.05 ± 0.54	6.93 ± 0.45	0.129
Side cut depth (μm)	16.31 ± 5.44	16.44 ± 5.91	0.889
Cap thickness (μm)	114.94 ± 7.53	115.50 ± 7.23	0.630
RST (μm)	318.89 ± 30.17	323.88 ± 36.96	0.351
Lenticule thickness (μm)	116.01 ± 27.78	118.35 ± 34.45	0.637

BCVA: best corrected visual acuity, CCT: central corneal thickness, N: number, SE: spherical equivalent, RST: residual stromal thickness. * denotes significant difference between the two groups.

**Table 2:** Postoperative visual acuity and spherical equivalent between the two groups.

Outcome	First KLEx Group (N = 80)	Second KLEx Group (N = 80)	*p*
UDVA (LogMAR)			
1 day	0.06 ± 0.08	0.09 ± 0.19	0.196
1 week	0.04 ± 0.04	0.03 ± 0.07	0.269
1 month	0.01 ± 0.03	0.00 ± 0.04	0.076
3 months	0.01 ± 0.02	0.01 ± 0.04	0.992
SE			
1 day	−0.31 ± 0.51	−0.38 ± 0.52	0.391
1 week	−0.39 ± 0.52	−0.45 ± 0.60	0.500
1 month	−0.44 ± 0.54	−0.43 ± 0.56	0.909
3 months	−0.47 ± 0.48	−0.41 ± 0.55	0.463

N: number, SE: spherical equivalent, UDVA: uncorrected distance visual acuity.

**Table 3:** Intraoperative and postoperative complications between the two groups.

Complication	First KLEx Group (N = 80)	Second KLEx Group (N = 80)	*p*
Intraoperative			
UIDPP	9	2	0.032 *
Incision tear	3	2	0.689
Cap perforation	0	0	>0.999
Residual lenticule	0	0	>0.999
Suction loss	0	0	>0.999
Postoperative			
Superficial punctate keratitis	0	0	>0.999
Dry eye disease	5	3	0.881
Corneal edema	0	0	>0.999
Interface foreign body	1	0	0.913
Epithelial ingrowth	0	0	>0.999
Diffuse lamellar keratitis	0	0	>0.999
Microbial keratitis	0	0	>0.999

N: number, UIDPP: unintended initial dissection of the posterior plane. * denotes significant difference between the two groups.

## Data Availability

The data used in the current study are available from the corresponding authors upon reasonable request.
